# Various Receipts

**Published:** 1856-02

**Authors:** 


### VARIOUS RECEIPTS.

The following we have seen recommended from time to time, and are perhaps worth a trial, as a proper preservation and proparation of food has an important bearing on health, while domestic conveniences preserve the temper of our wives and servants.

Much Honey from a little.—Those who wish to increase the quantity of their honey and also to increase its flavor, can do so by following Langstroth's directions, as follows:

"Dissolve two pounds of the purest white sugar in as much hot water as will be just necessary to reduce it to a syrup; take one pound of the nicest white-clover honey—any other light-colored honey, of good flavor, will answer—and after warming it, add it to the sugar syrup, and stir the contents. When cool, this compound will be pronounced, by the best judges of honey, to be one of the most luscious articles which they ever tasted; and it will be by almost every one preferred to the unmixed honey. Refined loaf-sugar is a perfectly pure and inodorous sweet, and one pound of honey will communicate the honey flavor to twice that quantity of sugar; while the new article will be destitute of that smarting taste which honey alone so often has, and will be found perfectly to agree with those who cannot eat the clear honey with impunity. If those engaged in the artificial manufacture of honey never brought any worse than this to market, the purchaser would have no reason to complain. As, however, the compound can be furnished much cheaper than the pure honey, many may prefer to purchase the materials and to mix them themselves. If desired, any kind of flavor may be given to the manufactured article. Thus it may be made to resemble, in fragrance, the classic honey of Mount Hymettus, by adding to it the aroma of the lemon balm, or wild thyme; or it may have the flavor of the orange groves, or the delicate fragrance of beds of roses, washed with dew.

---

Wintering Sweet Potatoes.—The 14th day of October, 1854, I dug about one-half bushel of sweet potatoes—packed them in two boxes—used dry plaster paris for packing, and placed them in a warm dry room, varying from 50° to 60°. On the 13th day of April, 1855, I planted them. Every one was sound and as good as in the fall. I have kept pumpkins and winter squashes one year in a warm dry room, and showed them at our annual fair, as sound as when severed from the vines. Dry sand may do as well.

---

Drying Pumpkins and Making Pies.—Cut them up and stew them till they are soft and dry; pound and strain them through a colander; then grease pie-pans, and spread it on a quarter of an inch thick, and dry it; roll it up, and put it away in a tight box or bag, from the insects. Every one of these rolls will make a pie. It is very easy now to make a pie. Put it in sweet milk, and let it soak about two hours; put in an egg, a tablespoonful of sugar, a teaspoonful of ginger, and one of allspice; and if you are lovers of pumpkin pie, as we are, you will pronounce it good.

Preserving Eggs fresh.—Make a barrel of lime-water as you would make white-wash, at least two weeks before you want to use it. Put your eggs in another barrel, stir up the lime-water well and pour it on the eggs, the lime settles around the eggs, and the water stands on the top of the lime, (the eggs all under lime.) Look at the barrel once in a while, to see if four inches of water, little more or less, covers the whole. If the water is all dried up, the lime gets hard, and they are difficult to take out when wanted, and you have to carry them somewhere else to wash off the lime; so always keep water on the top. Keep the vessels covered to keep out all dirt, or the eggs will look a poor, dingy color. Be careful about this in the lime and water, and you will have fine white eggs.

I cannot tell how long they will keep, as I never saw any spoil. I have some that are five years and a half old, as good as they ever were.

You may drop a few eggs in at a time as you get them fresh, but keep four inches of water on top. You can certainly test the goodness of each egg before putting in, by going into a dark room and having the egg between your eye and a candle, if sound the light will shine through with a reddish glow, if unsound the egg will look dark; reject every egg which has the least want of clearness.

---

Cheap Carpeting.—Sew together strips of the cheapest cotton cloth, of the size of the room, and tack the edges to the floor. Then paper the cloth with any sort of room paper. After being well dried give it two coats of varnish, and your carpet is complete. It can be washed like carpets without injury, retains its gloss, and on chambers or sleeping rooms, where it will not meet with rough usage, it will last two years as good as new.

---

To cook Potatoes.—Pare and put to soak in cold water from four to six hours; then drop into water which is already boiling—an essential point; a little salt added to the water improves them. Take them from the fire the moment they are done, pour off all the water and let them stand uncovered in the kettle over the fire till the water evaporates from the surface, and they are ready for the table.

Cheap and Healthy Diet.—Oat meal is excellent in porridge, and all sorts of cooking of that sort, and oat meal cakes are sweet, nutritious and an antidote for dyspepsia. Just now, we believe oats are the cheapest of any grain in market, and it is a settled fact that oats give the greatest amount of power of any grain consumed by man or beast. [This must be understood as referring particularly to Northern oats, as the majority of oats grown in Ohio, will not make good, sweet meal, suoh as we have tasted in Canada and Northern New York.—Eds. Cult.

Cracked wheat and loaf bread cost the same price or perhaps a less price for the wheat by the pound. A pound of the wheat, properly cooked, is worth more than four loaves of bread.

Hominy, samp, hulled corn, we have so often recommended and urged upon the attention of all, both rich and poor, as cheap, wholesome, nutritious food that we have induced many to try it, who would not give it up now under any consideration. We reiterate all that we have ever said in its favor. Thirty years' experience in its use only serves to confirm us in the opinion that it is such excellent and economical food that too much cannot be said in its favor. The only thing necessary in its cooking is to cook it enough—it cannot be cooked too much.

Every family should eat beans and peas, because of all articles they afford the most nutriment for the least money.

One bushel of white beans will feed more laboring men than eight bushels of potatoes. The beans will cost two dollars, the potatoes six.

A single quart of beans costs nine cents; a half-pound of salt pork, six cents; a pound of hominy, five cents; and that will give a meal to a larger family than a dollars' worth of roast beef, white bread, potatoes and other vegetables.

---

To Cook a Chicken.—Cut the chicken up, put it in a pan and cover it over with water; let it stew as usual, and when done make a thickening of cream and flour, adding a piece of butter, and pepper and salt; have made and baked a pair of short cakes, as for pie-crust, but rolled thin and cut in small squares. This is much better than chicken pie and more simple to make. The crust should be laid on a dish and the chicken gravy put over it while both are hot.

To Measure Grain and Corn in Bins.—To measure grain, multiply the width and length together, and that product by the height in cubic inches, and divide by 2.150, and you have the number of bushels.

To measure corn in the ear, find the cubic inches as above, and divide by 2.815, the cubic inches in a heaped bushel, and take two-thirds of the quotient for the number of bushels of shelled corn. This is upon the rule of giving three heaping half bushels of ears to make a bushel of corn. Some fall short, and some overrun the measure.

---

Curing Bacon without Smoke.—To smoke the best bacon, fat your hogs early, and fat them well. By fattening early you make a great saving in food, and well fattened pork. Then kill as early as the weather will allow, and salt as soon as the animal heat is gone, with a plenty of the purest salt, and about half an ounce of saltpetre to one hundred pounds of pork.

As soon as the meet is salted to your taste, which will generally be in about five weeks, take it out, and if any of it has been covered with brine, let it drain a little. Then take black pepper, finely ground, and dust on the hock as much as will stick, then hang it up in a good, clean, dry, airy place.

---

To Wash Clothes.—The night before washing-day, put the clothes to soak in water, and also place on the hot stove, in a suitable vessel, two pounds soap, cut small, one ounce borax, and two quarts water. These may be left to simmer till the fire goes out; in the morning the mixture will be solid. On washing-day, operations are commenced by setting on a stove or furnace the wash kettle nearly filled with cold water. Into this put about one-fourth of a pound of the compound, and then wring out the clothes that have been soaking and put them into the kettle. By the time that the water is scalding hot, the clothes will be ready to take out. Drain them well, and put them into clean cold water, and then thoroughly rinse them twice, and they are ready to be hung out. When more water is added to the wash-kettle, more soap should also be added, but the quantity needed will be very small.

Keeping Turnips, Parsnips, &c.—As late in the fall as is prudent to wait, take any old barrel, and put a good layer of dry leaves on the bottom, then put a layer of turnips or parsnips, then another course of leaves, and so alternating, being careful to put in a good supply of leaves between the roots and the barrel, and also between each course of vegetables.

Turnips properly put up in this way will not be *corkey*, will keep good all winter, and can be got at any time. Parsnips put up in this manner will be better in the winter and in the spring than if left in the ground, as is the common practice; besides you are not obliged to wait till the frost is out of the ground before you can have a mess. Your barrel of turnips should be kept in as cool a place as possible and still avoid freezing, as they grow unless kept dry and cool.

---

Buckwheat Porridge.—Take a quart of rich milk, and after boiling it hard, stir in as much buckwheat meal as will make it of the consistency of thick mush, adding one teaspoonful of salt and one of fresh butter. In five minutes after it is thick enough to take it from the fire. If the milk is boiling hard, and continues to boil while the meal is being stirred in, very little more cooking will be required. It should be placed on the table *hot*, and eaten with butter and sugar, or with molasses and butter. This is sometimes called a five minute pudding. It is excellent for children as a plain dessert, or for supper. Some add a seasoning of ginger or grated nutmeg before sending it to the table.

---

To Keep Grapes in Winter.—Let them hang on the vines as late as they can without freezing—pick in a dry day, place it in shallow boxes, not more than two clusters deep; keep it in as cool a place as you can and not let it freeze, and where there is sufficient circulation of air to carry off the moisture. I have kept them in this way until April, and though toward the last they were indented like raisins, they still retained their delicious flavor.

---

Mildew of Plants.—Sulphur and unslacked lime put into a tub of water, in which they are quickly and intimately mixed, then syringe with the clear liquid after these substances have settled at the bottom.

